# Best practices for cryo‐trapping time‐resolved crystallography with the Spitrobot crystal plunger

**DOI:** 10.1002/pro.70741

**Published:** 2026-09-15

**Authors:** Robert Bosman, Caitlin E. Hatton, Andreas Prester, Maria Spiliopoulou, Virginia Apostolopoulou, Friedjof Tellkamp, Pedram Mehrabi, Eike C. Schulz

**Affiliations:** ^1^ Institute of Biochemistry and Signal Transduction University Medical Center Hamburg‐Eppendorf Hamburg Germany; ^2^ Institute of Nanostructure and Solid State Physics University of Hamburg Hamburg Germany; ^3^ Center for Free‐Electron Laser Science CFEL Deutsches Elektronen‐Synchrotron DESY Hamburg Germany; ^4^ Institute for Nanostructure and Solid State Physics The Hamburg Centre for Ultrafast Imaging Hamburg Germany; ^5^ Max Planck Institute for the Structure and Dynamics of Matter Deutsches Elektronen‐Synchrotron Hamburg Germany

**Keywords:** Cryo‐trapping, Spitrobot, time‐resolved crystallography

## Abstract

Capturing meta‐stable conformations of enzymes and ligand complexes demands structural snapshots beyond static crystal structures. While time‐resolved serial crystallography at room temperature offers a time‐resolution down to the femto‐second domain, it requires large amounts of micro crystals, specialized beamlines, and considerable experience. Moreover, as the majority of enzymes display turnover‐times in the millisecond domain or slower, simpler methods can provide meaningful structural insight into enzyme catalysis. Vitrification of protein crystals can trap reaction intermediates by rapid cooling to <100 K and has traditionally been used to gain insight into long lived reaction intermediates such as product complexes. However, manual vitrification procedures are limited to long delay times of at least several seconds and heavily suffer from operator variability. A solution to this problem is provided by automatic crystal plunging devices, such as the *Spitrobot*, that plunge loop‐mounted protein crystals into liquid nitrogen within millisecond time‐scales. Versatile means of reaction initiation can be achieved either by micro dispensing a ligand droplet, or via optical excitation of light‐sensitive proteins, or via the photoactivation of caged compounds. In addition to the conceptual simplicity, another benefit of cryo‐trapping is that data can be collected at conventional synchrotron beamlines, exploiting their robust high‐throughput capabilities. Thus, compared to room‐temperature time‐resolved crystallography (RT‐TRX), users not only benefit from uncoupling sample‐preparation and data‐collection, but also from a reduction in the required technical expertise and ready access to radiation sources. However, as cryo‐trapping crystallography explores dynamic structural changes that become only visible by the comparison of several samples, experiments have to be carefully planned to carry out the necessary controls and to avoid mis‐ or over‐interpretation of the results. Here we describe a detailed protocol for cryo‐trapping time‐resolved crystallography using automated crystal‐plungers that enables researchers to map enzymatic reaction coordinate pathways within the millisecond domain.

## 1 INTRODUCTION

To understand the molecular foundations of life, it is imperative to understand the protein dynamics which underpin various biochemical functions. High‐resolution protein structures of meta‐stable functional states provide important information on ligand binding, cooperative mechanisms, allosteric regulation, detailed catalytic chemistry, and most crucially the interrelations between these phenomena. To understand enzyme chemistry this detail is required to fully interpret the reaction mechanism and the dynamic structural changes required to permit catalysis. Cryo‐trapping of intermediate species *in‐crystallo* is a powerful, yet conceptually simple technique in structural biology for answering these questions.

X‐ray crystallography provides protein structures at high spatial precision but these are typically static or at steady state. To overcome this, protein function can be initiated *in‐crystallo*, by, for example, photoactivation (Edman et al., 1999; Fedorov et al., 2003), or ligand diffusion (Hajdu et al., 1987; Fink & Ahmed, 1976; Gouet et al., 1996; Käck et al., 1998; Makinen & Fink, 1977; Molina et al., 2015; Schlichting et al., 1990; Schneider et al., 1998; Wilmot et al., 1999; Wilmot & Pearson, 2002); alternatively, perturbations such as pH jumps (Wilmouth et al., 2001) or release of solvated electrons (Berglund et al., 2002) can be utilized (Pearson et al., 2004). Cryo‐trapping then stops these reactions by rapidly cooling the sample to below the glass transition phase (~100 K) (Caramello & Royant, 2024; Moffat, 2001; Rasmussen et al., 1992). This works serendipitously with the common practise of cryo‐cooling samples to decrease radiation damage and increase crystal‐life times in the beam.

Cryo‐trapping thus enables structural characterization of meta‐stable states along enzymatic or photochemical reaction pathways. Historically, it has provided essential insights into a wide range of systems (Crosson & Moffat, 2002; Kouyama et al., 2004). Particularly, interrogating slow enzymes with turnover durations from minutes to seconds (Hekstra, 2023; Moffat & Henderson, 1995), extends our knowledge from apo, product, or dead‐end complexes to functional intermediates only present under out‐of‐equilibrium conditions.

Recently, serial crystallography, where partial diffraction patterns from thousands of micro‐crystals are merged, has allowed data collection with minimal radiation damage at non‐cryogenic temperatures (Chapman, 2019; Wilson, 2022). This has been expanded to time‐resolved studies with each crystal initiated separately for a given time delay. Once sufficient partial diffraction patterns have been collected, the delay time is shifted. This series of structures constructs a detailed set of structures across a reaction coordinate. Soon after this strategy was successfully applied at x‐ray free‐electron lasers, enabling serial‐femtosecond crystallography, it was also successfully adapted at synchrotron radiation sources for time‐resolved analyses of enzymatic reaction mechanisms (Weinert et al., 2019; Beyerlein et al., 2017; Coquelle et al., 2015; Gati et al., 2014; Mehrabi et al., 2019; Mehrabi et al., 2020; Prester et al., 2024; Stellato et al., 2014).

The adaptation to synchrotron beamlines was made possible by the development of microfocus beams and improved detectors, enabling extraction of useful signal from smaller crystals (Evans et al., 2011). Time‐resolved crystallography in general benefits from a reduced crystal size, which improves the homogeneity of reaction initiation. In photo‐triggered reactions, the crystal is more uniformly exposed to photons by reducing the absorption depth (Barends et al., 2024; Grünbein et al., 2020), while in ligand mixing experiments it shortens the diffusion time to the crystal centre and thereby improves the stoichiometry with the ligand (Makinen & Fink, 1977; Malla et al., 2023; Olmos et al., 2018; Pandey et al., 2021; Schmidt, 2013). For cryo‐trapping techniques, this reduced crystal size results in a more rapid equilibration than previously possible, enabling cryo‐trapping at time scales more akin to RT time‐resolved crystallography. The main advantages of time‐resolved cryo‐trapping experiments lie in their general simplicity, their wide applicability, and their compatibility with high‐throughput data collection. Rapid diffusion cryo‐trapping experiments have wide potential in structural biology, biochemistry, and mechanistic enzymology. Alternatively, they provide new opportunities for fragment screening, inhibitor design, and early‐stage drug discovery. The simplest version of the method requires only standard crystallographic tools; therefore, any MX laboratory can adopt the basic manual workflow.

Unique to cryo‐trapping experiments is, that the crystals size is also relevant to the vitrification time. Microcrystals in the range of 10–50 μm are typically optimal to minimize vitrification times, improving the overall homogeneity of reaction intermediates (Halle, 2004; Indergaard et al., 2025; Kriminski et al., 2002, 2003). Additionally, studies have demonstrated that significant heat removal occurs in the cold gas layer above the cryogen, and plunge speeds of ~1 m s^−1^ across a 2 cm gas gap can dominate the cooling process (Berejnov et al., 2006; Warkentin et al., 2006)—further complicating manual trapping experiments. Eliminating or minimizing this gas layer via automated approaches, thus significantly enhances vitrification speeds (Ryan, 1992). These consideration are unique to cryo‐trapping experiments making the time resolution and quality of trapping dependant on crystal size, sample environment, and plunge‐freezing conditions. However, recently developed high‐speed crystal plungers address these concerns, which enables trapping of enzymatic reaction intermediates in the millisecond time domain (Clinger et al., 2021; Indergaard et al., 2025; Kondo et al., 2025; Mehrabi et al., 2023; Spiliopoulou et al., 2025).

Commonly, RT and cryo‐trapping time‐resolved crystallography aim to determine protein conformational states and intermediates. Whether intermediates can be observed is fundamentally contingent on whether a non‐equilibrium state can be sufficiently populated *in‐crystallo*. This depends on the crystal form, an appropriate initiation strategy, the intermediate thermodynamics, and data collection strategy. Automated plungers such as the *Spitrobot* do not change these underlying biochemical or physicochemical determinants but facilitate measurements at time‐scales not accessible to manual cryo‐trapping. Additionally, they simplify and improve the sample handling and improve the measurement reliability. Given the growing adoption of time‐resolved crystallography, it is important to delineate how these results relate to cryo‐trapping results.

Cryo‐trapping has two distinctions to RT serial approaches. First, it permits a rotation data collection strategy via increased radiation tolerance thus improving structure factor approximation with current methods. Second, the rapid cooling can shift the protein conformational landscape toward lower entropy states, which can induce cryo‐cooling artifacts and increase sample heterogeneity due to the difference in freezing times (Halle, 2004). As such cryo‐trapping provides better data quality for more thermodynamically stable states, while serial approaches can capture higher‐energy, meta‐stable states that maybe quenched during freezing. This makes cryo‐trapping highly complementary to time‐resolved serial crystallography measurements (Bourgeois & Royant, 2005; Caramello & Royant, 2024). Therefore, the automated cryo‐trapping provided by automated plungers such as the *Spitrobot* improves the experimental methodology and workflow simplicity. This makes it a useful initial measurement before attempting more complicated serial experiments.

Here we summarize practical guidelines and protocols for sample preparation and data collection in cryo‐trapping time‐resolved crystallography. We initially focus on general considerations and important aspects in conventional (i.e., manual) cryo‐trapping. Later we focus our description on cryo‐trapping experiments using mechanical high‐speed crystal plungers, highlighting our recently introduced *Spitrobot* as an example. The *Spitrobot* is targeted at equipping biochemistry facilities with an automated crystal‐plunger platform to permit substantially shorter delay times and improved reproducibility, enabling mapping of enzymatic and ligand‐binding kinetics across millisecond delay times. The protocol stands to expand the user base for time‐resolved structural biology and enhance the mechanistic insights achievable in structure‐guided discovery pipelines. The workflow is applicable to mesophilic and thermophilic enzymes, multi‐domain proteins, and complexes of biomedical, industrial, or biotechnological relevance. Reaction‐initiation strategies include ligand delivery via droplet deposition or optical excitation of light‐sensitive systems. The protocol incorporates environmental control of temperature and relative humidity, enabling users to maintain crystal stability across diverse protein classes while fine‐tuning solvent content and lattice integrity.

## 2 PRELIMINARY EXPERIMENTS

**FIGURE 1:**
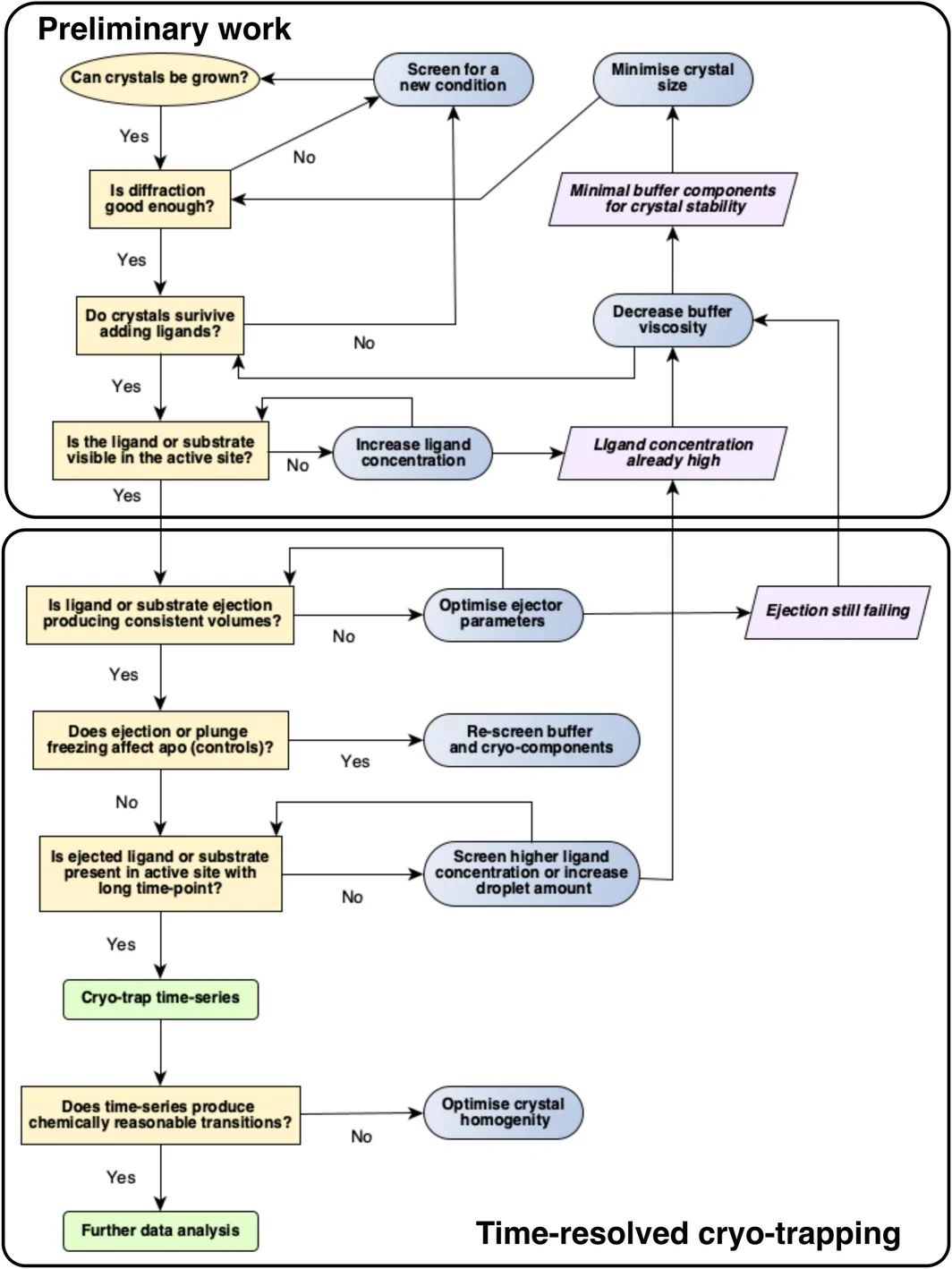
An overview of the experimental workflow. The work is split into two separate phases: the pre‐experimental work that includes sample optimization and confirmation of ligand binding, and the time‐resolved crystallography where a time‐series of structures is feasible under common conditions.

Several experimental choices and optimizations need to be addressed before proceeding to more complicated cryo‐trapping experiments, either as rapid manual or automated cryo‐trapping measurements. An overview and order of these considerations is provided in Figure 1.

### 2.1 Crystal growth and diffraction properties

As a central aim of the workflow is the comparison of multiple states at different delay times after reaction initiation, crystal growth has to be reliable and lead to comparable, high quality diffraction patterns. While the desirable resolution depends on the change to be observed, typically the resolution should not be lower than 2.5 Å. Obviously, any crystallographic pathologies like twinning heavily complicate the downstream data analysis and should be avoided.

**FIGURE 2:**
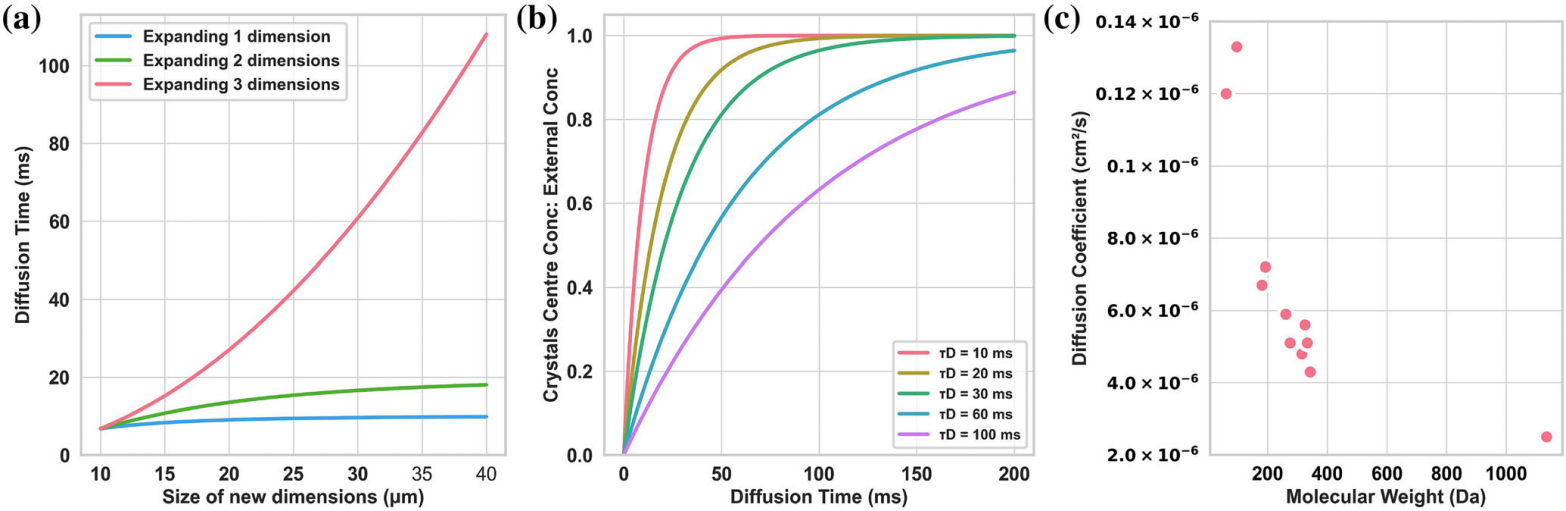
Diffusion considerations, calculations are adapted from Schmidt (2013), (a) graphs the effect of expanding crystals dimensions on the diffusion time ($\tau_{D}$) as defined in Schmidt (2013), with $D=5\times 10^{-6}$ ${\mathrm{cm}}^{-3}$. Note that this graph highlights that rod or plate shaped crystals can have typically long dimensions while diffusion is still relatively fast. (b) The ratio between the concentration at the crystal center and the external substrate concentration for different diffusion times. It is often desirable to use a high substrate concentration to reduce the diffusion time it takes to reach a given concentration. For example, as the graph indicates, if the desired concentration to achieve a good stoichiometry is 20% of the ligand concentration in the droplet, even with a long overall diffusion time (100 ms) it would take ~25 ms to reach the desired concentration. Therefore applying high ligand concentrations can in‐part mitigate slow diffusion kinetics. (c) The coefficients for several small molecules plotted against their molecular weight (data from (Geremia et al., 2006)). This plot highlights the correlation between molecular weight and faster diffusion coefficient and therefore a smaller molecule is generally preferable. There are caveats to this, such as charged ions, and we point the reader to more rigorous calculations and a discussion on diffusion into crystals provided in Geremia et al (Geremia et al., 2006).

While the initial manual experiments can be carried out using macroscopic crystals, it's imperative that time‐resolved experiments are carried out on crystals that permit homogenous reaction initiation, usually between 5 and 50 μm. Protocols for homogenous micro‐crystallization are identical to those required for RT‐TRX and have been previously addressed in focussed publications (Beale et al., 2019; Martin & Zilm, 2003; Shoeman et al., 2023; Stohrer et al., 2021). These micro‐crystallization conditions should be carefully optimized to achieve the best trade‐off in diffraction properties and reaction initiation, as these are fundamental limits to all downstream experiments. Figure 2a.

### 2.2 Cryo‐protection

Unlike room‐temperature time‐resolved crystallography, cryo‐trapping experiments typically require the addition of cryo‐protectants to prevent the formation of ice crystals and damage to the crystals during freezing (Garman & Doublié, 2003). These are typically organic solvents, sugars, or different molecular weight PEGs, all of which increase buffer viscosity. This poses a challenge for both optimal ligand diffusion and droplet injection. Furthermore, cryo‐protectants have been shown to bind to enzyme active sites, which makes structural interpretation challenging. An example of this is the binding of 2,3‐butanediol to xylose isomerase as shown in Figure 7b. An important control and part of prior screening is to test with only the addition of cryo‐protectant, both manually and with the *Spitrobot*, as outlined in Section 3. Therefore, cryo‐protectants should be optimized to reduce the concentration, utilize less viscous cryo‐protectants such as organic solvents or low‐molecular weight PEGs, and not interfere with the active site.

### 2.3 Viscosity effects

The above section addresses the use of cryo‐protectants, combined with already present buffer constituents to maintain crystal integrity. Crystallization solutions often have significantly different viscosities than aqueous water. There are several types of diffusion coefficients used to describe molecular motion in solution. Here we are concerned with the self‐diffusion coefficient, which describes how fast an individual molecule moves through the medium. The self‐diffusion coefficient of a small molecule can be estimated using the Stokes–Einstein relation (Supplementary information 1.1). For small‐molecule cryo‐protectants such as glycerol, the solution is homogeneous at the scale of the ligand. Glycerol is a small molecule, comparable in size to water, and mixes uniformly with it. The ligand therefore encounters a structurally uniform medium at all length scales. The dynamic viscosity is therefore the appropriate quantity to use in the Stokes–Einstein relation. It increases strongly with glycerol concentration, and tabulated values are available (Cheng, 2008).

For polymeric cryo‐protectants such as PEG, the behavior depends on both the concentration and molecular weight of the polymer. At low concentrations, polymer coils are well separated and the solution is approximately homogeneous, so the dynamic viscosity can be used directly in the Stokes–Einstein relation. As concentration increases, chains begin to overlap. The concentration at which this starts to occur is called the overlap concentration c*, and it marks the boundary between the dilute and semi‐dilute regimes. Both the value of c* and the bulk dynamic viscosity depend on the molecular weight of the polymer: higher molecular weight PEGs reach c* at lower concentrations and produce more viscous solutions at a given concentration. In the semi‐dilute regime, the bulk dynamic viscosity becomes strongly concentration‐dependent and increases rapidly with concentration. Moreover, PEG forms a transient network characterized by a correlation length ξ. Small ligands with hydrodynamic radius *RH* smaller than ξ are expected to experience a local micro‐viscosity significantly lower than the bulk dynamic viscosity (Holyst et al., 2009). In this regime, the dynamic viscosity therefore represents an upper bound on the effective viscosity experienced by the ligand, and the Stokes–Einstein estimate of the diffusion coefficient should be treated as a lower bound.

In practice, the Stokes–Einstein relation with tabulated viscosity provides a straightforward and conservative estimate of how cryo‐protectant concentration affects ligands' diffusion. We provide practical calculations (Supplementary information 1.1) intended to give the user a way to estimate approximate ligand diffusion times through a crystal under a given set of conditions.

### 2.4 Soaking experiments

Once apo protein crystals can be grown and give sufficient data quality with an optimized cryo‐protectant, manual soaking experiments are essential to confirm substrate binding. These follow standard practices in crystallography, with ligands added directly to the crystallization well or fished into droplets with cryo‐protectant and ligand prior to flash‐freezing. A key reason for utilizing faster, more precise cryo‐trapping methodologies is that enzymatic turnover is often faster than can be trapped manually. Both rapid turnover and an occluded active site produce an empty active site. In this instance, biochemically assaying the protein crystals can confirm catalysis within the lattice (Makinen & Fink, 1977). Reducing the crystal size decreases the equilibration times, Figure 2a, alternatively, soaking at lower temperatures can slow the reaction enough to confirm binding and turnover (Hajdu et al., 1987; Makinen & Fink, 1977). Ensuring that a product state is observed via manual soaks acts as an essential positive control for ligand mixing experiments. If no evidence of activity in crystals is detected, then re‐screening for a new crystal form is required.

### 2.5 Substrate and ligand considerations

When considering ligand addition reactions there is an additional choice of initiating ligand or substrate. As the *Spitrobot* enables mixing times which would be manually unfeasible, it permits working with native substrates that catalyze too quickly for manual cryo‐trapping. This minimizes the need to work with mutants which often lack key chemical groups at important residues that would otherwise predominate the native pathway. Despite this, diffusion is often a limiting factor within the confines of the crystal lattice. In this instance the associated enzyme kinetics dictate whether intermediates build up sufficiently to be observed *in‐crystallo*.

Early treatments of this question (Makinen & Fink, 1977) focused on the shortest crystal dimension, the critical depth, beyond which substrate diffusion becomes rate‐limiting, since reducing the crystal size improves the substrate‐to‐enzyme stoichiometric ratio and shortens the overall diffusion time. The critical depth itself can be enlarged by lowering the catalytic efficiency, by both decreasing *K*_cat_ and increasing *K*_*m*_. This is a sensible strategy if the aim is to measure enzyme kinetics *in crystallo* without having to account for diffusion effects. The objective of a time‐resolved crystallography experiment is different, however, as a sufficient intermediate population needs to build up *in‐crystallo*.

*K*_*m*_ is defined as *K*_off_ plus *K*_2_ divided by *K*_on_, and is therefore a ratio of the rates that affects the [ES] concentration. Consider, that if all else remains the same doubling a substrates *K*_*m*_ will double the concentration required *in‐crystallo* to achieve a given enzyme‐substrate occupancy at a certain time‐point. This is also important when ligands have low solubility, if the *K*_*m*_ is also high it may become difficult to achieve sufficient binding and occupancy even during long soaking. Typically, the experimenter can at least find reported values for *K*_*m*_ and *K*_cat_ for a given substrate or these can be extracted from kinetic data. When working with fast enzymes utilizing substrates with a slower turnover and a low *K*_*m*_ can allow sufficient intermediate build‐up *in‐crystallo*. Alternatively, if the question being addressed requires a given substrate, a slower enzyme homolog can provide an alternative. When there is flexibility over which substrates or ligands to use, there are several preferred characteristics: high solubility, high diffusion coefficient, a slower turnover rate, and a low *K*_*m*_, to ensure sufficient intermediate build‐up. Ultimately, testing different ligands experimentally is a sound initial approach and the time‐resolved cryo‐trapping method outlined here is a simple, efficient way to perform these initial experiments Figure 2.

### 2.6 Delay times

Assuming that an observable product‐state has already been achieved via manual soaking, different strategies can be used for selecting delay times. If *in‐crystallo* kinetics are known (e.g., assessed spectroscopically), it is straightforward to start with suitably populated delay times. If *in‐crystallo* kinetics are unknown, delay times can be decreased from the initial control following a quasi‐logarithmic spacing to a few‐hundred milliseconds (Mehrabi et al., 2020). Initial data‐analysis should indicate when events are occurring *in‐crystallo* and further delay times can be planned accordingly.

## 3 CONTROL EXPERIMENTS

**FIGURE 3:**
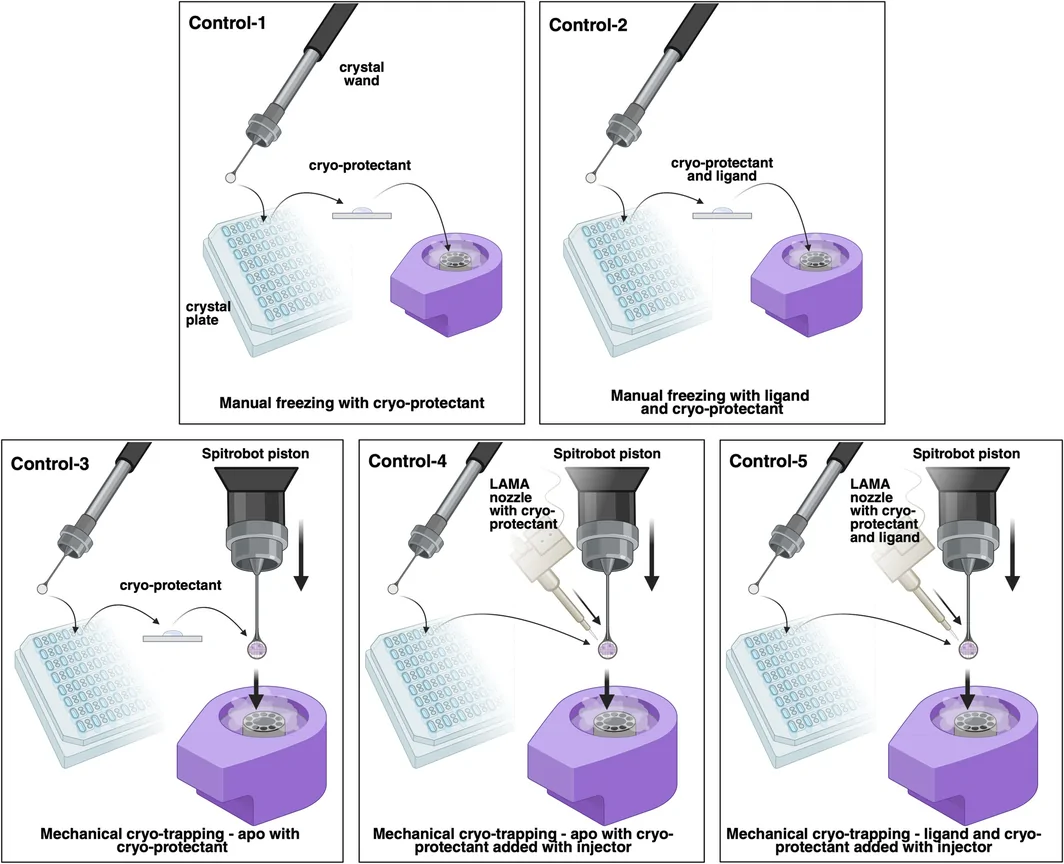
Schematic overview of the controls. Control‐1, manually freezing the sample with cryo‐protection should ensure that the cryo‐protectant is sufficient as usual in crystal screening, but uniquely whether the cryo‐protectant binds or may interfere with the time‐resolved study. Control‐2 is a soak with the substrate. If the substrate is not present, re‐screening is likely required. Control‐3, the sample can be mounted normally, but spitting is excluded. If the plunge freezing is causing adverse effects, it should be visible here. Control‐4, the spitter is used to add cryo‐protectant rather than a soak before mounting. Ideally Control‐1, 3, and 4 will produce identical maps. Finally, Control‐5 is essentially a long time‐point measurement. This should produce similar results to manual Control‐2.

Before moving from manual to automated cryo‐trapping, several control measurements are important to ensure that changes can be correctly attributed to the added ligand (Figure 3). These controls are focused around ensuring the automated cryo‐trapping process is not interfering with the samples. In contrast to canonical single‐crystal structure determination experiments, it is vital to carry out these controls for cryo‐trapping experiments. For a meaningful evaluation of the data, it has to be verified that any observed electron density changes originate from a biochemical function and are not simply experimental artifacts due to the perturbation of the crystals. These controls adequately apply to any automated plunge freezing device and are recommended initial measurements on the *Spitrobot*.

### 3.1 Control‐1: Manually frozen with cryo‐protectant

Collecting an unperturbed apo state without change to the sample and an optimized cryo‐protectant is the initial baseline measurement. Establishing a coherent baseline can be achieved by measuring multiple crystals to similar data quality. This point is also ideally suitable for the selection of an appropriate crystal mount, discussed in further detail in section (Supplementary information 1.2).

### 3.2 Control‐2: Manually frozen with ligand or substrate and cryo‐protectant

Ligand or substrate can be added and cryo‐trapped manually. The most straightforward approach is transferring the crystal into a well containing the ligand and freezing in liquid nitrogen after several seconds. This is essentially the soaking experiment that is a key part of the preliminary work. We should see a product state or evidence of binding and catalysis in the active site. Please refer to Section 5.1 for a detailed outline of the procedure.

### 3.3 Control‐3: Mechanical cryo‐trapping—Apo samples with cryo‐protectant

Prior work details that the method of freezing can change the characteristics of the sample (Berejnov et al., 2006). Therefore, when starting an automated cryo‐trapping approach, controls should be measured to ensure that utilizing the device recapitulates the data quality and electron density from original apo states. Crystal damage due to insufficient humidity, temperature control, or the rapid plunge freezing process should be detected from this control. Additionally, the higher vitrification speed provided by automated plungers may reduce the required cryo‐protectant concentration.

### 3.4 Control‐4: Mechanical cryo‐trapping—Apo samples with cryo‐protectant added via systems liquid application method (LAMA nozzle)

Other sources of error can come about from distortions caused by adding the cryo‐protectant with the ligand via droplet addition (Berejnov et al., 2006) as it can be difficult to ensure the same total volume of cryo‐protectant compared to manual soaks. As highlighted previously, the cryo‐protectant may bind the active‐site Figure 7b, particularly if the cryo‐protectant concentrations differ. Therefore, control measurements are required to characterize any effects caused by cryo‐protectant addition with LAMA as opposed to manual cryo‐protection.

### 3.5 Control‐5: Mechanical cryo‐trapping—Ligand and cryo‐protectant via droplet addition

This series of controls will be able to confirm that interpreted density is caused purely by ligand addition. Therefore the final control is to re‐capitulate the ligand complex observed with the manual soaks established before proceeding to automated cryo‐trapping using the plunge freezing device. An important consideration here is that the ligand stoichiometric ratios are in a similar regime to ensure comparable maps.

## 4 TIME‐RESOLVED CRYO‐TRAPPING WITH THE *SPITROBOT*

The previous sections addressed a series of general considerations and required preparatory work before attempting time‐resolved cryo‐trapping experiments. We will now give a brief introduction to the *Spitrobot* system (Kondo et al., 2025; Mehrabi et al., 2023; Spiliopoulou et al., 2025), which we use as an example for automated cryo‐trapping devices used in this protocol.

The *Spitrobot* utilizes the LAMA method to enable accurate and reproducible cryo‐trapping experiments with versatile time resolutions and the use of SPINE standard pins for compatibility with most synchrotrons (Mehrabi et al., 2019). It is equipped with a humidity flow device (HFD), which supplies a constant air flow at a preset temperature and humidity, allowing for multi‐temperature crystallography and a suitable protein crystal environment. The effect of structural changes during flash‐cooling (Halle, 2004) is reduced due to the rapid and repeatable plunge‐freezing of the *Spitrobot* compared to manual flash freezing (Spiliopoulou et al., 2025).

The *Spitrobot* setup comprises three principal parts. The *Spitrobot‐2* device, the ancillary devices, and a driving computer. The main device as outlined in Spiliopoulou et al. (2025) internally integrates the following principal components; (i) a standard‐pin mount on a motorized piston, (ii) a mounting stage with XYZ motion for the Autodrop pipette, (iii) a sample shutter system, (iv) a LN_2_ dewar mount, (v) a puck rotation and alignment system, (vi) two camera's with XY alignment stages for sample visualization and validation images. This devices also houses the electronics for coordinating the devices during automated cryo‐trapping. The device is principally controlled by three buttons located on the front panel. Triggering the device is done with buttons, B1 and B2, on the panel above the sample position, this is a safety feature to ensure the experimenters hands cannot interfere with the mechanism during plunging. The third button, B3, to the right of the sample position is designed to cycle between a sample visualization mode by providing diffuse background light, puck alignment mode by switching a guide beam, and neither state. It is also pressed to reset the *Spitrobot* from its error state (Figure 6c).

**FIGURE 4:**
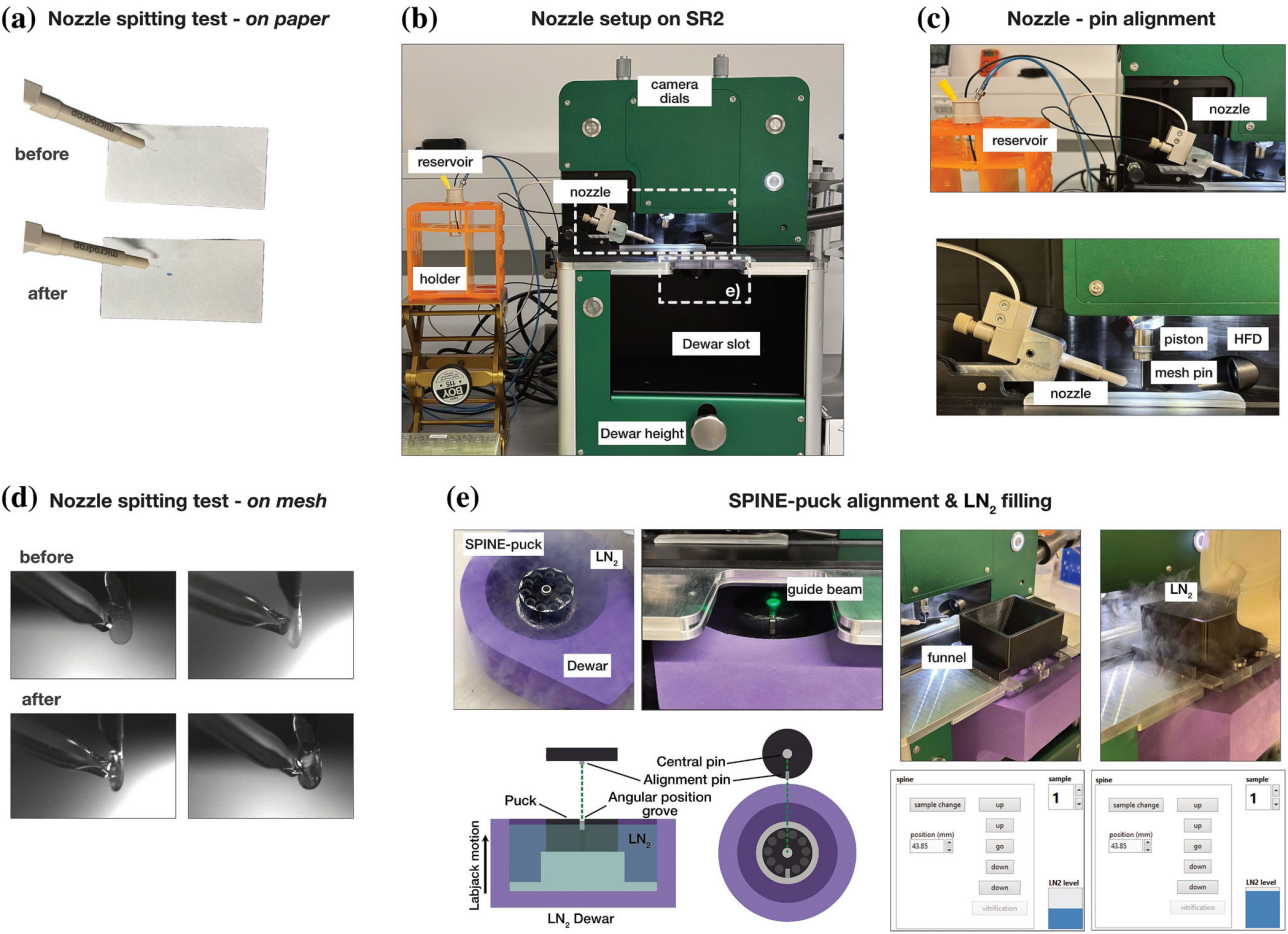
*Spitrobot* setup, (a) before and after successful ejection from the Autodrop pipette assessed using Wator paper. This water sensitive paper turns into a strong blue on contact with water and therefore gives a clear indication of successful ejection. The nozzle can then be mounted on the *Spitrobot‐2*; (b) the nozzle points into the sample position from the left, while the HFD nozzle is postioned orthogonally. In order to maintain an appropriate pressure in the tubing the buffer reservoir is mounted on a jack that can be adjusted accordingly (Supplementary Table 1). The sample piston positions the sample vertically between both (c). Once the Autodrop pipette and the sample mesh are aligned on camera (d) different ejection volumes should be tested aiming to achieve a thin film of ligand sample that covers the mesh. (e) The SPINE puck is cooled in a custom holder that permits the puck to rotate. The rotation mechanism sits above the dewar and an alignment pin locks into the angular positioning groove. A central pin fits into the pucks centre position. By manually moving the dewar on the lab‐jack, the centre position and pin can be aligned, with an assisting guide light, while the alignment pin is aligned by eye. Once both are in position the lab‐jack underneath the dewar can be raised and locks the centre position and alignment pin into place. The puck can then be rotated by the mechanism above the dewar which has a manual handle on the outside of the *Spitrobot*. The puck is thereby mounted into the *Spitrobot*, a step‐by‐step guide is provided in the protocol section.

The two main ancillary devices are the HFD, and a triggering system, principally the Autodrop pipette (LAMA nozzle) from microdrop technologies GmbH for which an in‐built mount is incorporated (Figure 4b,c). Prior to performing experiments the ancillary devices need to be setup and mounted to the *Spitrobot‐2*. Since, the Autodrop pipette ejection parameters need to be re‐optimized for a new buffer, as drop‐ejection parameters change with the buffers physiochemical properties, we outline a specific Autodrop ejection optimization protocol (Supplementary Figure 1). Separately, the HFD needs to be equilibrated to the desired temperature and humidity approximately 30 min before running the *Spitrobot*. Recent work has incorporated an optical trigger system (Kondo et al., 2025) currently positioned via a custom mount to where the ejector sits. Finally, a control laptop is used to set the experimental parameters and monitor the LN_2_ level. Delay times for camera triggering, pin‐mount position height, sample numbering, validation image output folders, and sample delay time are all specified via a LabView interface (Mehrabi et al., 2023) from the laptop.

## 5 PROTOCOLS AND PROCEDURES

### 5.1 Manual cryo‐trapping protocol

There are multiple versions of manually adding ligand and rapidly plunge cooling crystals. Here we outline a simple version for a microcrystal slurry that mimics the process achieved on the *Spitrobot* and therefore works well for controls 1 and 2. We would expect that a 10 s soaking time would be achievable. If product or end‐states are already well explored crystallographically then skip to Section 5.2.1.

1. Prepare the liquid nitrogen foam dewar and a puck as usual.
2. Mount the micromesh onto a magnetic wand, place the Whatman paper on the surface below the underside of the micromesh.
3. Pipette 0.25–1 μL microcrystal slurry onto the frontside of the mesh, aiming to achieve a visible meniscus.
4. Refill the pipette with 0.25–1 μL containing ligand or buffer solution and hold the pipette tip just above the frontside of the mesh. Move with the mesh in the next steps, keeping it just above the frontside.
5. Gently pivot the magnetic wand on the rim of the base, until the underside of the mesh contacts the whatman paper and absorbs the majority of the liquid. Raise the mesh just off the blot paper.
6. Pipette the ligand or buffer solution and repeat step 5.
7. Immediately, or after the pre‐requisite time, plunge the mesh into liquid nitrogen.

### 5.2 *Spitrobot* setup and configuration

Prior to working with the *Spitrobot*, the two principal ancillary devices need to be optimized and then configured. A detailed outline to optimize the droplet ejection, followed by setting up the Autodrop pipette and HFD, is presented in Supplementary information 1.3 and Supplementary information 1.4, respectively. Once all the ancillary devices are set up and mounted, the *Spitrobot‐2* device can be set up.

#### 5.2.1 Setting up and mounting SPINE puck

8 *Critical*: Ensure the SpearLab foam dewar and puck holder are completely dry. Residual liquid left in the puck holder will freeze the puck to the holder and prevent rotation. Residual liquid can also transform into floating ice crystals that can adhere to samples and compromise data collection.
9 Place the puck into holder and fill with liquid nitrogen. Once the puck has stopped boiling away liquid nitrogen, using the cryovial tongs, rotate the puck manually to check the mechanism is still operational.
10 Align the puck such that the angular positioning groove points toward the foam dewar handle. Place the foam dewar onto the inbuilt labjack.
11 Press the B3 button until the puck alignment light turns on (a green light that illuminates the center position for the puck). Align the puck center to the green light (Figure 4e).
12 Rotate the sample dial until in sample position “7.” Align the angular positioning groove with a corresponding spoke located on the underside of the heating plate. Raise the labjack until both the puck center and sample dial spoke are connected.

#### 5.2.2 Starting the *Spitrobot*

13 Ensuring the *Spitrobot* is powered on, start the control software. Once the *Spitrobot* has finished its internal checks. Request a shutter “open,” and shutter “closed” to ensure the *Spitrobot* is properly connected.
14 *Recommended*: Open the shutter and check the puck position matches the sample dial, rotate the puck to confirm the puck mechanism is rotating the puck (Figure 6c).
15 Rotate the puck to sample position 1.
16 If the Autodrop is not already mounted, mount the Autodrop pipette in the holder opposite the HFD and reconfirm sample spitting. Move the Autodrop pipette to the retracted position for more space around the piston.

### 5.3 Cryo‐trapping samples with the *Spitrobot*

We outline the core procedure for automated plunge freezing using the *Spitrobot*; we assume that the *Spitrobot* is already setup and configured. The HFD should be equilibrated to the set temperature and humidity, the SPINE puck should be loaded into the *Spitrobot* and set to position 1, with the Autodrop pipette already mounted.

*Recommended*: Test plunging and spitting on an empty loop, running a test plunge on an empty loop after setup ensures the machine is working as expected (Figure 4d).

*Recommended*: For each solution it is important to optimize the amount of substrate (number of droplets) deposited as excess ligand solution will contribute to background, while insufficient ligand produces inconsistent mixing. We aim for a uniform coverage across the mesh but without causing a rounded meniscus. This can also be carried out when testing buffers offline as outlined in Supplementary information 1.3.

#### 5.3.1 Setting the cryo‐trapping parameters

17 In the LabView menu request the delay time in milliseconds.
18 Using the dials, manually align the nozzle in the *X*, *Y*, *Z* direction.
19 Returning to the LabView menu, set the pre‐camera time delays to the number of milliseconds before the sample is plunged that the cameras should record images. For long time points, this can remain 200 ms; at time points <200 ms, a minimum of 25 ms before plunge is recommended.

#### 5.3.2 Sample mounting and alignment

20 There are two principal ways to mount samples depending on whether they are prepared in batch or are grown in a plate. The mounting procedure has small but important differences.
  - Microcrystals grown in batch

- Mount the micromesh to the *Spitrobot* piston.
- Align the cameras so the micromesh is visible on the left side of the image and in focus
- Rotate the mesh so the concave side faces the Autodrop pipette.
- Pipette 0.25–1 μL of microcrystal slurry onto the front of the mesh.
- Blot the convex mesh side with Whatman paper until only residual liquid remains.
  B Crystals fished into loops from plates

i Fish the crystal(s) from the wells and quickly mount to the *Spitrobot* piston to minimize drying out.
ii Align the cameras so the loop is visible on the left side of the image and in focus.
iii Rotate the loop so that it is face onto the Autodrop.
iv Carefully blot‐crucially without touching the crystal, remove as much mother liquor to leave a thin counting over the crystal(s).

21 Detach the Autodrop pipette from its retracted position and move forward onto the right side of the camera view. Use the Autodrop pipette *XYZ* stage to position the pipette tip above, pointing down toward the center of the sample mount (Figure 4c,d).
22 Align the Autodrop pipette to be inline with the sample, looking down the length of the pipette; adjust the pipette tip. The pipette tip should also come into focus of the camera.

#### 5.3.3 Triggering the *Spitrobot*

23 Depress the two trigger buttons located on the front panel of the *Spitrobot* with both hands to initiate plunge freezing. If plunge freezing needs to be halted, release the trigger buttons. The buttons on the front panel will turn red to indicate the process has been halted (Figure 6b).

- *Caution*: The freeze plunging mechanism is driven by an electric motor with a piston that is capable of piercing skin with the sample pin. The trigger buttons are designed to be pushed with both hands to prevent the experimenters' hands getting in the way of the mechanism while plunging. Others assisting with sample preparation should also stand back from the device while plunging.

**FIGURE 5:**
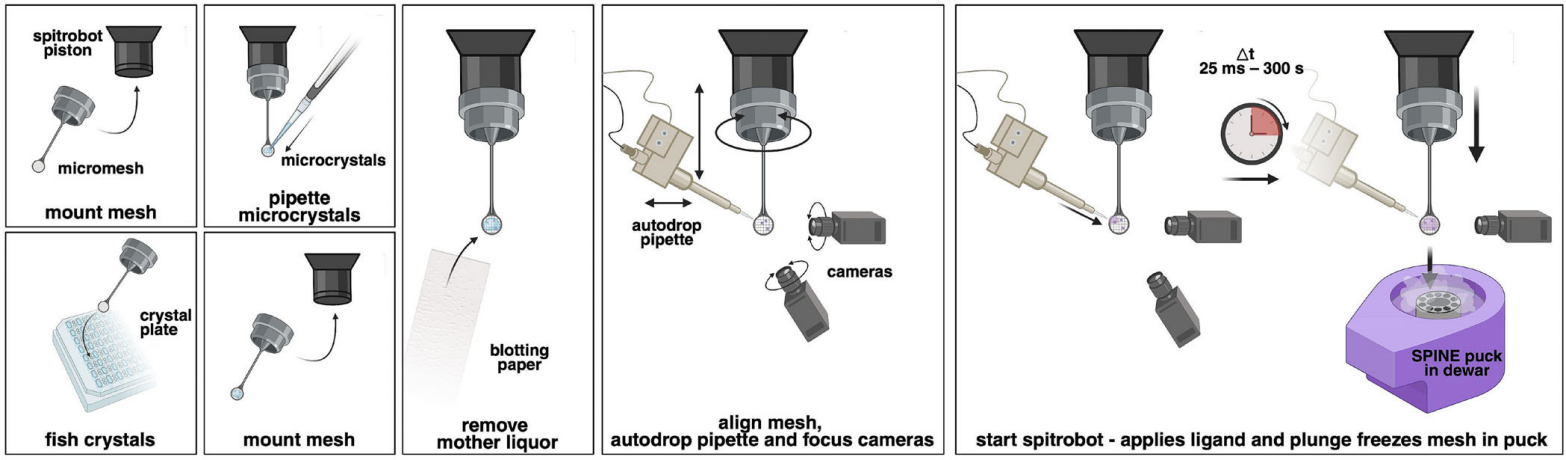
Schematic overview of the automated cryo‐trapping workflow. Crystals can be pipetted directly onto a MicroMesh from a slurry or fished from a crystallization tray. Excess mother liquor should then be removed by blotting from the back side of the mesh. The mesh face with the deposited crystals is positioned in front of the Autodrop pipette, which is aligned closely with it. The *Spitrobot* then automatically triggers the Autodrop pipette to apply ligand solution and vitrifies the sample in liquid nitrogen at the set delay time.

24 *Spitrobot automated rapidly cooling process*: The automated vitrification takes place as outlined in previous publications (Mehrabi et al., 2023; Spiliopoulou et al., 2025). In brief, the cameras take an image of the sample a set time before liquid deposition. The Autodrop pipette will deposit ligand. The camera will take another image to confirm deposition. If the time‐point is short, the shutter will open prior to these steps, and the sample will be immediately plunged; for longer time‐points, the shutter will open after deposition. The sample will remain in the down plunged position for several seconds before the magnet releases the sample into the puck. The plunger will then return to its home position, and the shutter will close. Several issues can occur during this process, as outlined in Figure 5, a full guide on how to handle these can be found in the Troubleshooting table (Supplementary Table 2).
25 Confirm droplet deposition via images taken before and after Autodrop triggering before continuing to the next sample.

#### 5.3.4 Resetting the *Spitrobot*

**FIGURE 6:**
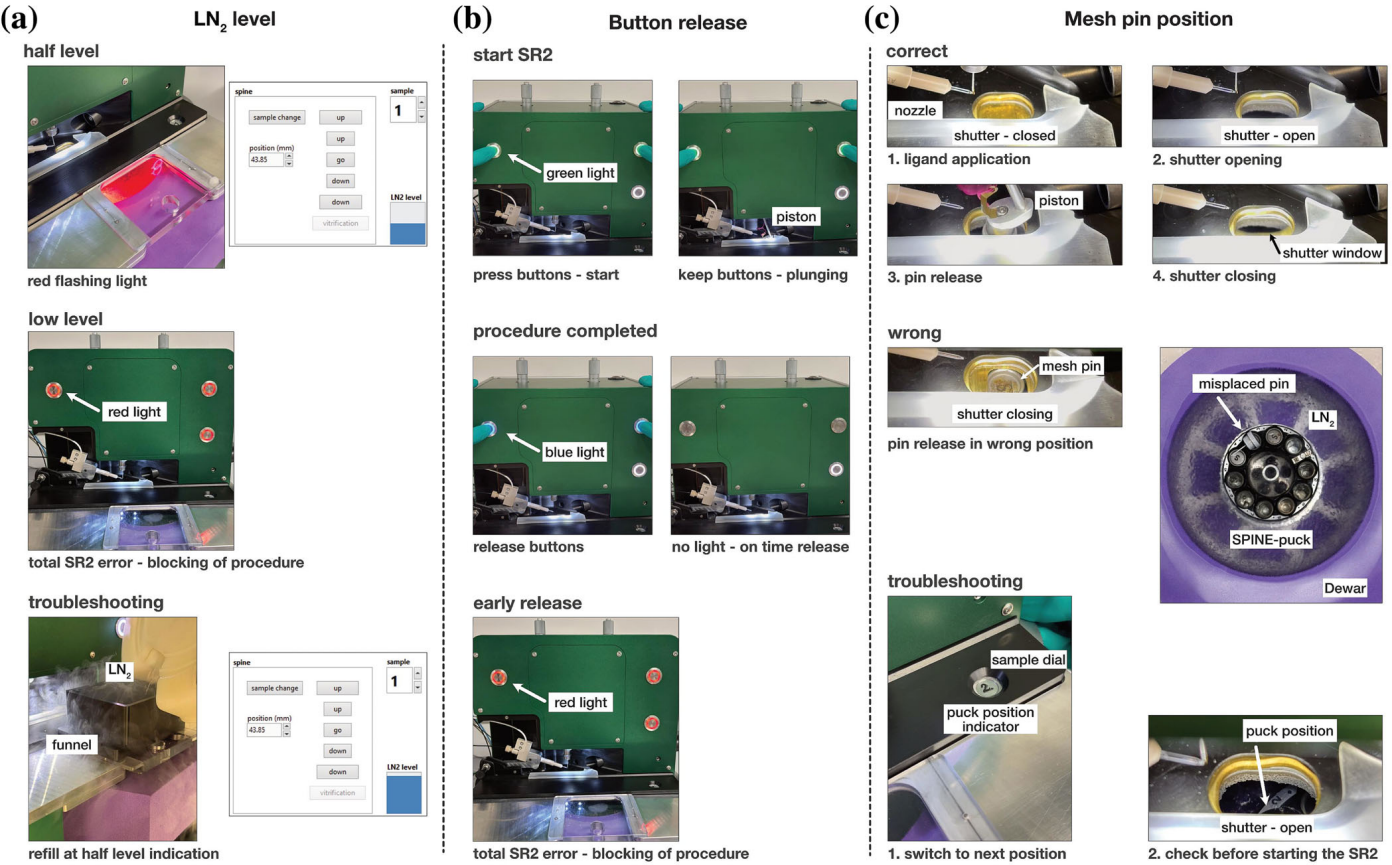
*Spitrobot* troubleshooting. (a) Low LN_2_ causes a constant drift in the freezing profile as LN_2_ boils away adding to delay time variation, which particularly effects sub‐second delay times. The *Spitrobot‐2* includes an automatic detection for the reduction in LN_2_, when the LN_2_ is low an initial flashing stage warns the experimenter. If the LN_2_ falls below a defined minimum all buttons turn red at which point the *Spitrobot* will not begin the automated vitrification procedure, until the LN_2_ is replenished. (b) Once the trigger buttons have been depressed they must be maintained until the vitrification cycle has completed indicated by the buttons turning blue, this is a deliberate safety feature. Releasing the triggers halts the procedure and the *Spitrobot* has to be reset before further vitrification can proceed. A mechanical complication (c) upon vitrification can cause the pin to by misplaced either by being trapped in the shutter mechanism or not correctly entering the SPINE puck.

26 Rotate the puck dial to the next sample position. It is recommended to manually open and close the shutter to confirm the mechanism has rotated for the first few samples (Figure 6c).
27 Check the liquid nitrogen fill gauge, if it is no‐longer at maximum fill using the LN_2_ funnel. If this gauge falls below a certain level while mounting or aligning the next sample it will prevent triggering plunge freezing until the LN_2_ is refilled while the sample is mounted. This should be avoided as LN_2_ boiling off from the funnel can cool the sample despite the stable environment provided by the HFD.
28 Move the Autodrop pipette back a few millimeters with the stages before pulling it back to the retracted position and fixing it with the nozzle pin.
29 For preparing samples for a new delay time repeat from step 17, if the delay time remains the same, repeat from step 20 and mount a new *Spitrobot*. If the puck is complete, either because it's full or no more samples are required, the foam dewar can be removed and the puck stored in a transport dewar for shipping to the beamline. Loading a new puck is outlined in Section 5.2.1.

### 5.4 Data‐collection strategies for cryo‐trapped samples

Data collection for *Spitrobot* samples follows the same procedures already developed for modern MX‐beamlines (Cipriani et al., 2006). Guidelines and protocols on how to optimize data collection on modern x‐ray source have been extensively addressed elsewhere (Garman & Sweet, 2007; Holton, 2009; Popov & Bourenkov, 2003; Winter et al., 2019). There are specific considerations for data collections from microcrystals deposited on micromeshes. In this case there are three potential data‐collection strategies. Option A, crystals are collected as rotation datasets, for samples with ice or extensive residual ligand solution that can present difficulties with alignment; Option B, multiple rotation wedges collected on separate crystals and merged together; or Option C, the micromesh can be rastered to collect a cryo‐SSX dataset.

#### 5.4.1 Rotation datasets from micromeshes

*Option A: Full rotation datasets*

- Mount the micromesh and align the face perpendicular to the beam; at this stage the crystals are either clearly visible or, as often with microcrystals, may be difficult to visualize. If crystal positions are clear, align and collect a rotation dataset; otherwise, gridscan as outlined below.
- Select a small beam size and raster the mesh; most beamlines have this capacity and can display the grid scan as a heat map of diffraction containing images.
- For single crystals clearly visible in the heatmap, align the beam to this position and adjust the beamsize to crystal size; otherwise, align to well diffracting areas and sculpt the beamsize as guided by the grid scan.
- If the sample thickness makes it impossible to align the crystal orthogonally to the mesh, perform a second grid‐scan at 90° to ascertain the crystal depth on the grid. Use both to align the crystal.

*Option B: A series of wedge dataset collections*

- Repeat steps (i)–(iii) from Option A.
- Rather than a full rotation, at each strong diffraction point collect a small rotation (5°–30°). These can be scaled and merged into a single dataset using XDS (BLEND) or dials.multiplex (Foadi et al., 2023; Gildea et al., 2022; Kabsch, 2010).

#### 5.4.2 Cryo‐SSX

*Option C: rastered serial dataset collections*

- Mount the micromesh and align the face perpendicular to the beam, utilizing a microfocus beam if possible or a smaller beam size to raster the micromesh.
- Rotate the mesh 30° and repeat (i), rotate the sample back the other way 60° and repeat (i). This should minimize preferential orientation and increase the total number of lattice orientations sampled. Separate geometries may need to be refined for each mesh orientation during data processing if the rotation significantly changes detector distance.

## 6 ANTICIPATED RESULTS

**FIGURE 7:**
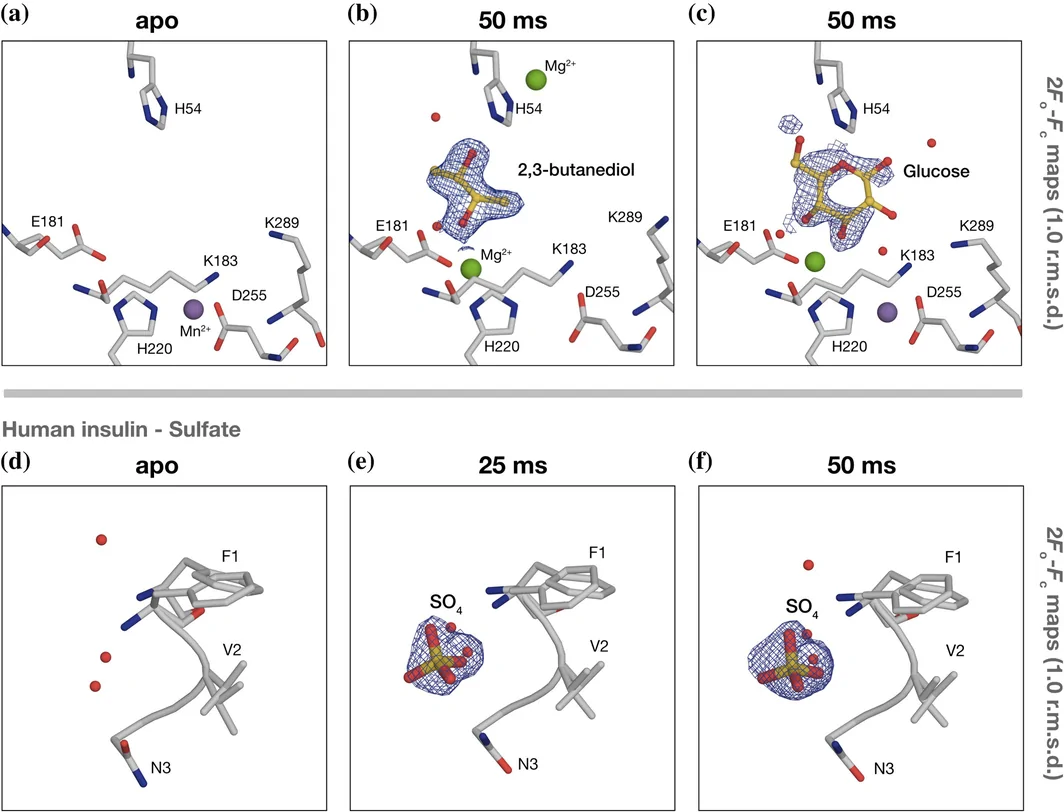
Results from a cryo‐trapping experiment on xylose isomerase and human insulin microcrystals. 2*mF*_*o*_–*DF*_*c*_ maps contoured to 1 r.m.s.d of (a) the empty apo active site (PDB code: 9R45 (apo state) and (b) the initial control experiment where only the cryo‐protectant 2,3‐butanediol was present in the buffer (PDB code: 8AWY). The control revealed that the cryo‐protectant bound in the active site, and outcompeted the ligand, therefore a different cryo‐protectant was optimized for the subsequent experiments. This allowed the unambiguous assignment of (c) the glucose ring after 50 ms (PDB code: 9R47 (50 ms). Human insulin microcrystals were triggered with low pH buffer supplemented with 1 M Na_2_SO_4_ and collected as single crystal rotation data. 2*mF*_*o*_–*DF*_*c*_ maps contoured to 1 r.m.s.d of the active site show the presence of the SO_4_ (PDB codes: (d) 9R48 (apo state), (e) 9R49 (25 ms), and (f) 9R4A (50 ms)). The data and figure are adapted from (Mehrabi et al., 2023; Spiliopoulou et al., 2025).

Automated cryo‐trapping experiments will produce at least one puck for control data and then 5 samples per delay times.* These can be transferred to unipucks to meet the beamline requirements. Sufficient cryo‐protection should produce a clear droplet where crystals can be visualized or aligned via x‐ray centring procedures. 2*mF*_*o*_–*DF*_*c*_ electron density maps for delay times should show electron‐density for the positive control and this can be tracked as delay times are shortened. Provided the underlying physical chemistry discussed in the experimental design is amenable to millisecond mixing the presence of ligand should be observed into this regime Figure 7.

### 6.1 Data analysis

Approaches to analyze cryo‐trapping data are no different to strategies for time‐resolved work in general. A standard 2*mF*_*o*_–*DF*_*c*_ map should reveal significant intermediates. For comparability a standard $R_{\text{free}}$ set should be maintained. Absolute value END‐RAPID maps ensure datasets are on a comparable scale for interrogating changes between delay times (Lang et al., 2014). For earlier delays where ligand occupancy is anticipated to be low, initial density is often observable in OMIT or POLDER‐OMIT (POLDER) maps before 2*mF*_*o*_–*DF*_*c*_, and is therefore particularly helpful in delineating the early stages of ligand binding although polder maps are generally helpful across all time‐points (Liebschner et al., 2017). It is typical to calculate a difference density or *F*_*o*_–*F*_*o*_ map for photo‐triggered time‐resolved studies. The calculation of *F*_*o*_–*F*_*o*_ maps has been applied appropriately to cryo‐trapping on rhodopsins. This can be useful in mixing experiments, however coordinated waters or ions are often present in the active sites and are therefore subtracted against the ligand. Therefore, *F*_*o*_–*F*_*o*_ maps often have less utility in interpreting active‐site chemistry. Instead they are best for assessing the structural changes to the protein and high‐occupancy non‐dissociating co‐factors present in the apo or ground state that undergo changes during the reaction. A straightforward approach is to calculate all these once a model is reasonably well refined to cross‐check consistency between maps. This provides a rigorous assessment of different maps which should provide a reasonable assessment of changes seen in the data.

Several strategies have been developed to analyze time‐resolved data. Despite the best experimental setup, the data will contain a mix of transient states. Therefore, a key goal in time‐resolved data analysis is to deconvolve the mixed states into a reasonable reaction pathway which is traversed by the protein. We can define these roughly into two camps: (a) multi‐state modeling methods or (b) decomposition methods.

Multi‐state modeling methods try to directly quantify the extent of the mixture by refining against the data and deriving intermediate populations and chemical conformations from this refinement procedure. This typically requires prior assumptions about the constituents of the data. Decomposition methods deploy a mathematical breakdown to define how many intermediates are present and attempts to extract or calculate the populations and a map for each state, this approach usually works best considering the time‐series as a whole. We use this categorization to give a framework to the different approaches, which are not contradictory and can be applied simultaneously to interrogate a given time‐series.

Initial time‐resolved mixing experiments modeled the most obvious state that could be observed in a 2*mF*_*o*_–*DF*_*c*_ map as done for at equilibrium measurements. Early light triggered experiments performed on chromophore containing photo‐responsive proteins used a two state model with a starting state (A, either apo or “dark” state) and end state (B), refining the occupancy for each A or B state across the time‐series (Pande et al., 2016; Nango et al., 2018; Bosman et al., 2025; Oda et al., 2018). Once a dark‐state model is refined this approach is practically implemented by adding a second state or b‐state, either covering the entire protein or only features delineated from *F*_*o*_–*F*_*o*_ maps. This model is then refined against the light state data, only refining the second states position. Occupancies are usually allowed to vary within a constrained range, determined either by complimentary data or chemical feasibility. This could be extended to calculate extrapolated maps whereby occupancy estimates are used to extrapolate structure factors and maps for a full occupancy state. This is a non‐trivial back‐calculation for which the program Xtrapol8 (De Zitter et al., 2022) provides a robust framework. More recent work has leveraged multi‐state refinements to model several chemical states into each time‐point and track the occupancy and B‐factors associated with each state (Prester 2026). This is a convenient approach although it requires either a plausible set of models, or can be used to test the compatibility of hypothetical reaction pathways within the data, with the later requiring a robust assessment of an unrealistic state.

Decomposition methods in their simplest form perform an SVD on the data to extract the main components at each time‐point and fit a reaction mechanism to the overall experiment (Schmidt et al., 2003). This can also be used to calculate intermediate maps from the components that are extracted and thereby provide an intermediate state map from mixed data (Vallejos et al., 2024). Further extensions have deployed neural networks to assess and extract intermediate maps and notional kinetic data (Biener et al., 2024). This approach assumes that the mathematically derived time‐independent features are chemically coherent and interpretable, which, given the low signal‐to‐noise in time‐resolved data, can often prove challenging. Currently, multi‐state methods are the most commonly used; however, new and more integrative approaches are under development. This includes RoPE to assess the underlying torsion angle changes from the derived models (Ginn, 2023), and integrative multi‐state models providing new ways to analyze varying heterogeneity across time or perturbation series, although they have yet to be applied to time‐resolved data (Hancock et al., 2025; Keedy et al., 2015).

## 7 CONCLUSIONS

As the importance of dynamic structural biology grows, the value in studying proteins undergoing catalysis will increase. As such, utilizing regular and more advanced cryo‐trapping approaches to provide additional structural data on protein functional states provides a promising direction for research. The protocol outlines considerations for when and how to apply this methodology, both as a very simple bench‐top measurement or utilizing automated cryo‐trapping as a straightforward and relatively robust way to reliably reach delay times unachievable manually. The *Spitrobot* is an automated cryo‐trapping device which has been specifically designed to facilitate these measurements as a standard bench‐top device for use alongside other commonly used biochemical tools. This envisages a point when time‐resolved crystallography, either as a time‐resolved room temperature serial measurement or a more widely accessible time‐resolved cryo‐trapping measurement, will be centerpiece datasets for an integrative structural biology approach to studying protein dynamics.

## AUTHOR CONTRIBUTIONS

**Maria Spiliopoulou:** Visualization; writing – review and editing. **Andreas Prester:** Conceptualization; visualization; writing – review and editing; writing – original draft. **Virginia Apostolopoulou:** Formal analysis; methodology. **Robert Bosman:** Writing – original draft; visualization; writing – review and editing; conceptualization. **Friedjof Tellkamp:** Resources; software; methodology. **Caitlin E. Hatton:** Conceptualization; writing – original draft; visualization; writing – review and editing. **Eike C. Schulz:** Conceptualization; funding acquisition; writing – review and editing; visualization. **Pedram Mehrabi:** Conceptualization; funding acquisition.

## FUNDING INFORMATION

The authors gratefully acknowledge the support provided by the Max Planck Society. PM acknowledges support from the Deutsche Forschungsgemeinschaft (DFG) via grant No. 451079909 and from a Joachim Herz Stiftung add‐on fellowship. ES acknowledges support by the Federal Ministry of Education and Research, Germany, under grant number 01KI2114. Funded by the European Union. Views and opinions expressed are, however, those of the author(s) only and do not necessarily reflect those of the European Union or the European Research Council Executive Agency (ERCEA). Neither the European Union nor the granting authority can be held responsible for them.

## Supporting information


**FIGURE 1.** Overview workflow for optimizing nozzle ejection. The process is a series of optimizations to deal with buffer viscosity and high solute concentration.
**Table 1**. Materials, reagents and equipment.
**Table 2**. Troubleshooting overview.

## Data Availability

The data that support the findings of this study are openly available in Protein Data Bank (PDB) at https://www.rcsb.org, reference number SCR_012820.

## Footnotes

* As a rule‐of‐thumb, 5 samples should be prepared per time‐point and at least 3 should be collected for cross‐comparison.
