# Supplementary material for: Best practices for cryo‐trapping time‐resolved crystallography with the Spitrobot crystal plunger
Source: Protein Sci. 2026 Sep 15;35(10):e70741. doi: 10.1002/pro.70741 (PMC13575983; doi:10.1002/pro.70741)
Supplement: Supplementary file 1 [file PRO-35-e70741-s001.pdf]

1 Supplementary Information

1.1 Effect of viscosity on ligand diffusion

For a small molecule, self-diffusion coefficient can be estimated using the Stokes-Einstein relation:

$$D = \frac{k_B T}{6\pi\eta R_H} \tag{1}$$

Where k_B is Boltzmann's constant, T is temperature (C°), R_H is the hydrodynamic radius of the diffusing ligand, and η is the dynamic viscosity of the medium. The Stokes-Einstein relation is valid when the diffusing molecule is larger than the solvent molecules, such that the solvent can be treated as a continuous medium. This condition is expected to be mostly satisfied for small ligands diffusing through water, glycerol-water mixtures, or PEG solutions. The viscosity contribution from the protein and buffer components is close to that of water and is negligible compare to the cryo-protectant contribution. The dynamic viscosity η is therefore the key parameter governing how cryo-protectant addition affects ligand diffusion. We outline two examples using cryo-protectants common in *Spitrobot* experiments: glycerol (Case 1) and PEG 400 (Case 2).

1.1.1 Case 1: Indole in 30% v/v glycerol, room temperature

The dynamic viscosity of the glycerol water mixture at a given concentration and temperature is calculated using the empirical formula from Cheng (2008)[1]. Converting 30% v/v glycerol to a mass fraction gives $C_m \approx 0.35$, using the density of glycerol $\rho_g = 1277 - 0.654T\,\mathrm{kg\,m^{-3}}$[1], which gives $\rho_g \approx 1261\,\mathrm{kg\,m^{-3}}$ at 298 K. The dynamic viscosity of the mixture at 298 K is then $\eta_{\mathrm{glycerol}} \approx 2.58 \times 10^{-3}$ Pa s. The hydrodynamic radius of indole is estimated from the molecular volume, assuming the molecule is approximately spherical[2]:

$$R_H = \left(\frac{3M}{4\pi N_A \rho}\right)^{1/3} \tag{2}$$

Where M is the molecular weight, N_A is Avogadro's number, and ρ is the molecular density. Using $M = 117.1\,\mathrm{g\,mol^{-1}}$ and $\rho = 1.22\,\mathrm{g\,cm^{-3}}$ (values from[3]), we obtain $R_H \approx 0.34$ nm. We note that this is an approximation; the molecule is not perfectly spherical, and a more accurate value would require a direct measurement of the diffusion coefficient of indole in water.

The self-diffusion coefficient of indole in pure water at $T = 298$ K, where $\eta_{\mathrm{water}} = 8.9 \times 10^{-4}$ Pa s, is:

$$D_{\mathrm{water}} = \frac{k_B T}{6\pi\eta_{\mathrm{water}} R_H} \approx 7.4 \times 10^{-10}\,\mathrm{m^2\,s^{-1}} \tag{3}$$

The self-diffusion coefficient in 30% v/v glycerol is then:

$$D_{\text{glycerol}} = \frac{k_B T}{6\pi \eta_{\text{glycerol}} R_H} \approx 2.6 \times 10^{-10}\,\text{m}^2\,\text{s}^{-1} \tag{4}$$

This corresponds to a ratio $D_{\text{glycerol}}/D_{\text{water}} \approx 0.35$, indicating that 30% v/v glycerol reduces ligand diffusion to approximately 35% of its value in pure water. The diffusion time into a crystal is estimated using the analytical solution to

Fick's second law for a shoebox crystal[4]:

$$\tau_D = \frac{4}{D\pi^2}\left(\frac{1}{a^2} + \frac{1}{b^2} + \frac{1}{c^2}\right)^{-1} \tag{5}$$

Where a, b, and c are the crystal half-dimensions. For a cubic crystal with $a = b = c = 10\,\mu$m, the estimated diffusion times are $\tau_{D,\text{water}} \approx 18$ ms and $\tau_{D,\text{glycerol}} \approx 52$ ms. Glycerol molecules are comparable in size to small ligands ($R_{H,\text{glycerol}} \approx 0.35$ nm[5], $R_{H,\text{indole}} \approx 0.34$ nm), which approaches the limit of validity of the Stokes-Einstein relation. However, it has been experimentally demonstrated that the Stokes-Einstein relation remains applicable for molecules of this size, provided the appropriate viscosity of the medium is used[5]. The bulk dynamic viscosity may slightly overestimate the local viscosity experienced by the ligand, and the actual diffusion time is therefore likely shorter than 52 ms.

1.1.2 Case 2: Chloroacetate in 30% w/v PEG 400, room temperature.

PEG 400 is a low molecular weight polymer that behaves closer to a small-molecule co-solvent than a typical polymer. To determine which diffusion regime applies, we first estimate c^*. This can be done from the intrinsic viscosity $[\eta]$, which is defined as the contribution of the polymer to the solution viscosity in the dilute limit, and depends only on the polymer and solvent, not on concentration. For PEG in water, an empirical expression for $[\eta]$ as a function of molecular weight is available[6]:

$$[\eta] = 2.0 + 0.016 MW^{0.76}\ \text{mL g}^{-1} \tag{6}$$

The overlap concentration is then estimated as[7]:

$$c^* = \frac{1}{[\eta]} \tag{7}$$

For PEG 400, this gives $[\eta] \approx 3.5$ mL g^{-1} and $c^* \approx 28\%$ w/v. A concentration of 30% w/v PEG 400 therefore sits at the boundary between the dilute and semi-dilute regimes, and the dynamic viscosity remains a reasonable approximation for estimating ligand diffusion. For higher molecular weight PEGs, c^* is reached at much lower concentrations. For example, PEG 4000 reaches c^* at approximately 9% w/v[8]. In those cases, the micro-viscosity argument becomes relevant.

The hydrodynamic radius of chloroacetate is estimated using the same molecular volume approach as in Case 1 using equation (2) with M = 94.5 g mol^{-1} and ρ = 1.58 g cm^{-3} for chloroacetic acid [4], we obtain $R_H \approx 0.27$ nm. As with Case 1, we note that this is an approximation. Chloroacetate is typically used in its anionic form in solution, which may slightly increase the effective hydrodynamic radius due to hydration of the charged group.

The self-diffusion coefficient in pure water at T = 298 K was estimated in Case 1 and is $D_{\text{water}} \approx 9.1 \times 10^{-10}$ m^2 s^{-1}. For 30% w/v PEG 400 at 298 K, the dynamic viscosity is $\eta \approx 3.24 \times 10^{-3}$ Pa s, interpolated from tabulated values[9]. Since 30% w/v PEG 400 sits at the boundary of $c^* \approx 28\%$ w/v, the solution is approximately in the dilute regime and the dynamic viscosity is a reasonable approximation for the viscosity experienced by the ligand. The self-diffusion coefficient in the cryo-protectant solution is then:

$$D_{\text{PEG}} = \frac{k_B T}{6\pi\eta_{\text{PEG}} R_H} \approx 2.6 \times 10^{-10} \text{ m}^2 \text{ s}^{-1} \tag{8}$$

This corresponds to a ratio $D_{\text{PEG}}/D_{\text{water}} \approx 0.28$, indicating that 30% w/v PEG 400 reduces ligand diffusion to approximately 28% of its value in pure water. We use the same relation from Schmidt[4] as in Case 1 to estimate the diffusion time into a modelled crystal. For a cubic crystal with $a = b = c = 10\,\mu$m, the estimated diffusion times are $\tau_{D,\text{water}} \approx 14$ ms and $\tau_{D,\text{PEG}} \approx 50$ ms. Both cases show a consistent, roughly three-times increase in diffusion time relative to pure water. In contrast to glycerol, the PEG 400 estimate is subject to an additional source of uncertainty: as the concentration approaches c^*, the viscosity experienced by the ligand may deviate from the bulk value, and the diffusion time should be treated as an upper bound.

1.2 Crystal mount selection

Provided that a loop can be mounted onto SPINE style magnetic base, it is compatible with the *Spitrobot*. While large macrocrystals can be fished in nylon or Kapton loops, micro-crystals are best loaded onto Micro-mounts, or Micro-Meshes. Standard crystal fishing considerations apply here, such as tailoring the loop size to the crystal. The *Spitrobot* is compatible with regular SPINE mounts large (500 μm) or small (5-40 μm) crystals of different morphologies can be easily mounted as for standard data-collection. Crystals can be fished directly from crystal plates into nylon or Kapton loops, while microcrystals in slurries are typically pipetted directly on Kapton meshes with remaining mother liquor removed via blotting.

1.2.1 Micromeshes

Micromeshes are preferred for mounting microcrystals grown in batch. Slurries can be directly pipetted onto micromeshes. Crystal densities are 10^4 and excess mother liquor easily removed via blotting with the mesh preventing crystals being removed

by the blotting paper. Hundreds to thousands of crystals can be mounted simultaneously allowing the mesh be rastered for a cryo-SSX strategy, or rotated on individual crystals depending on experimental requirements. As a solid support, meshes can also be glow-discharged to better spread the crystal slurry or ligand solution across the mesh.

1.3 Optimising the Autodrop pipette for ejection

Crystallisation buffers have different constituents and therefore viscosities and this effects ejection, therefore prior to spitroboting the buffer should be optimised. In-order to form controlled pL-nL droplets we utilize a piezoelectric injector, similar to other rapid mixing systems[10,11,12]. These ejection strategies are sensitive to the buffer viscosity, for example, the *Spitrobot* injector is inconsistent when large molecular weight PEGs are present at over 20% (v/v). As such, the injection parameters must be optimized for each buffer.

1. Fill the Autodrop pipette reservoir with water, flush the line with water to confirm there are no blockages.
2. Attach the Autodrop pipette and flow water through the nozzle to establish a laminar flow.
3. Refill the reservoir with buffer, and repeat 1-2 with the new solution.
4. Start with Autodrop pipette single pulse mode, voltage=160, pulse width=20, pulse delay=9. Around 1000Hz and 200-300 drops. This should deposit around 19 nL.
5. If the sample ejects repeatably then no further work is needed. If no droplet is observed then the flow diagram lays out the iterative optimisation.
6. Once reliable droplet ejection has been established re-clean the lines with water and EtOH.

1.4 Setting up ancillary devices

1.4.1 Setting up the Humidity Flow Device (HFD)

The HFD device provides humidity and temperature control at the sample point and needs to be equilibrated and mounted prior to starting experiments. Most protein crystals survive at 20°C and 95 % humidity which is the assumed default, unless crystals are grown under different conditions (4°C) or where pre-explored environmental conditions are desired[13]. Start the HFD 30 minutes before sample preparation to allow time for equilibration.

1. Using an Allen key unscrew the HFD hose port on the front of the *Spitrobot*, fit the HFD nozzle into the port and position 5-10 cm from the sample position, re-tighten the set screw.

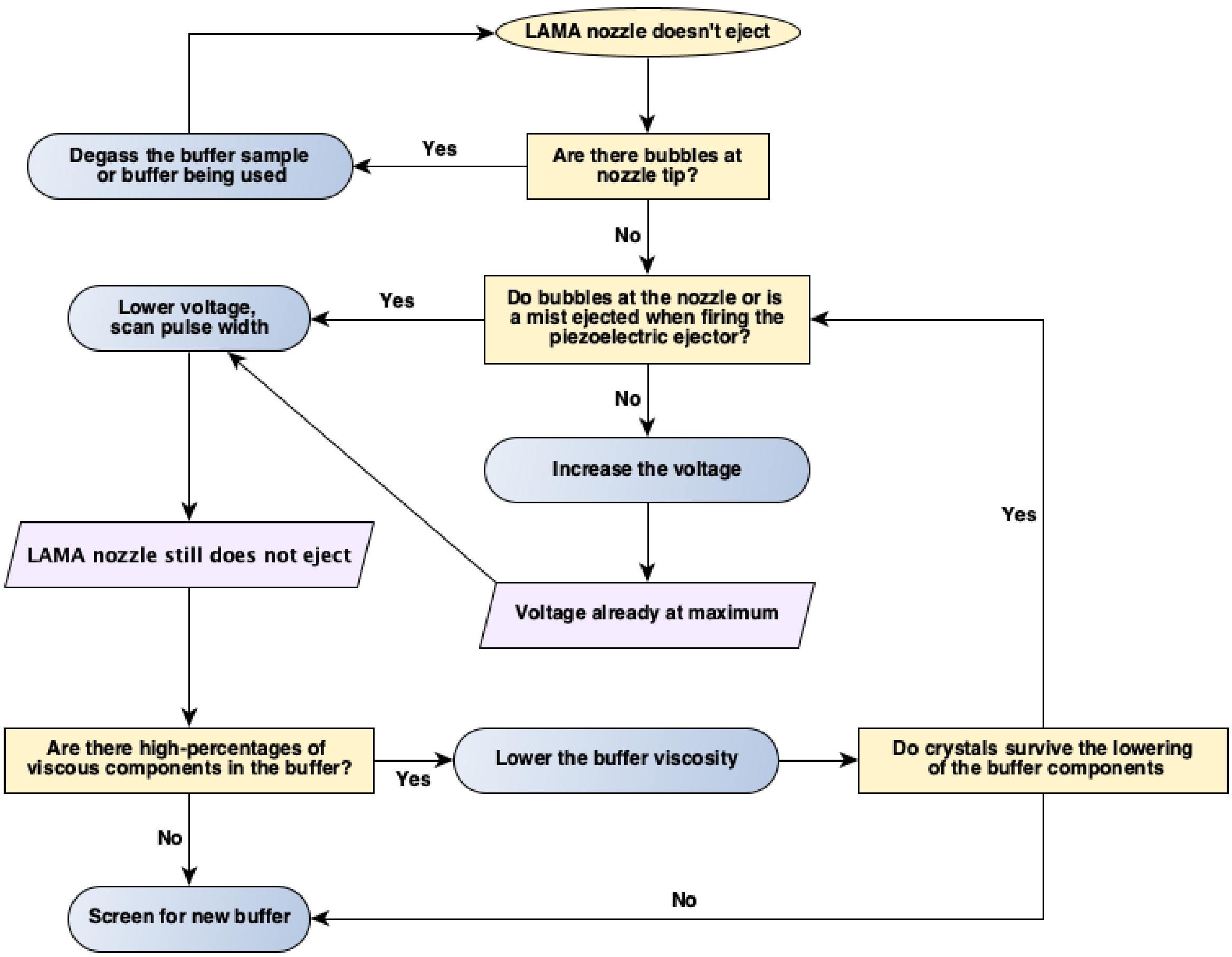

FIGURE 1 Overview workflow for optimising nozzle ejection. The process is a series of optimisations to deal with buffer viscosity and high solute concentration.

2. Attach a compressed air-line to the back of the HFD. Confirm airflow by adjusting the 'humid air' and 'dry air' flow meters to pre-calibrated requirements for temperature and humidity.
3. Fill a 60 mL syringe with deionised water and attach to the HFD water port, fill the waterbath until above the minimum volume as indicated on the side.
4. Using the display set the HFD to 'running', and navigate to the designated panel to set the 'des' values to the required temperature and humidity.
5. Place a hand in front of the HFD nozzle to confirm that humidified air is being produced.
6. Wait for around 15 - 30 minutes for the temperature and humidity to equilibrate to the required values, if this seems to be taking longer troubleshoot as in (**Table 2**).

1.4.2 Autodrop pipette configuration - Confirm droplet formation

The Autodrop pipette needs to be flushed with the appropriate spitting solution (buffer or ligand), spitting confirmed, and mounted to the device:

1. Fill the Autodrop pipette reservoir with water, flush the line with water to confirm there are no blockages.
2. Attach the Autodrop pipette and flow water through the nozzle to establish a laminar flow.
3. Refill the reservoir with ligand containing buffer (or buffer if performing controls), and repeat 1-2 with the new solution.
4. Adjust the reservoir height to ensure a slight over pressure at the pipette tip.
5. Using the piezo-injector controls, manually trigger a single droplet, visualise this on the water sensitive paper **(Figure 4a)** if this fails troubleshoot as in **(Table 2)**.
6. Once single droplets are established trigger a droplet burst for the desired deposited volume, repeat this 3-4 times to confirm consistency.
7. Leave the droplet nozzle in a humid environment to prevent the tip drying up while setting up the *Spitrobot*.
8. **PAUSE POINT**: If the HFD is already mounted and running, the Autodrop injector can be mounted and left in the humidified stream to prevent buffer evaporation and corresponding crystallisation of buffer components, blocking the injector **(Figure 4b)**.

2 Materials

TABLE 1 Materials, Reagents and Equipment

Item	Purpose/Description	Vendor/Order Number
**Reagents**		
Protein crystals	either in plates or as batch slurry	
Crystal buffer		
Crystal buffer with cryo-protectant		
Ligand in appropriate buffer with cryo-protectant		
Liquid nitrogen		
Isopropanol, 70% filtered		
Deionised water, filtered		
**Equipment**		
Wator paper	For confirming ligand droplet size and distribution	Macherey-Nagel, 90610
Whatman blotting paper	For wicking excess liquid off crystals	Whatman, WH5201
Magnetic wand	or similar products used for crystal fishing	Mitegen, MW-1C
MicroMesh^{TM}	The *Spitrobot* accommodates anything on a SPINE mount, for blotting away excess mother-liquid and mounting microcrystals; MicroMeshes act similarly to serial chip devices	Mitegen, 700/25, 400/10-50
Goniometer bases	any base mounts compatible with SPINE pucks and the intended beamline goniometer can be used here	Mitegen, B5 SPINE style
P1 Pipette	specifically for pipetting small volumes onto mesh grids	
CryoVial Tongs	for sample manipulation	Mitegen, M-R-1013195
Puck CryoTongs	for puck manipulation	Mitegen, M-CP-111-030
Spearlab Foam dewar		FD-800
**Equipment – *Spitrobot***		
Automated plunge freezing device		*Spitrobot*
Autodrop pipette	Nozzle diameters: 30 μm, 50 μm or 70 μm	microdrop Technologies, AD-KH-501
Microdrop controller		microdrop Technologies GmbH
Puck rotation device	insert for FD-800, standard foam dewar	
Humidity Flow Device (HFD)		
60 mL syringe		Eppendorf
Compressed air line		
Spine pucks [14]		

3 Troubleshooting

TABLE 2 Troubleshooting overview

System	Problem	Possible cause	Solution
**General**	Sample is not applied evenly over the mesh	Inaccurate pipetting, buffer constituents	Glow discharge samples
	Samples display significant ice formation upon data collection	Deposited sample too thick for cryo-protectant to be effective	Reduce deposited droplets or increase cryo-protectant concentration
	No ligand in the active site	See overview table and experimental design	See overview table and experimental design
**HFD**	HFD humidity not stabilising	Incorrect combination of dry and humid airflow	Check settings; if correct, lower both dry and humid air
	HFD not able to reach requested humidity	Too much dry air flow compared to humid air flow	Lower the dry airflow or raise humid airflow
		HFD hose bent preventing passage of air	Straighten HFD nozzle
	HFD consistently reading too high humidity	Too much humid air flow compared to dry air flow	Lower the humid airflow or raise dry airflow
	HFD not able to reach temperature	Room temperature higher than desired temperature	Switch on chiller
		HFD hose bent preventing passage of air	Straighten HFD nozzle
	No detectable airflow from the HFD	HFD hose bent preventing passage of air	Straighten HFD nozzle
		Water bath low	Refill water bath with 60 ml syringe
		No air flow to HFD	Check airflow inlet nozzle
**Autodrop pipette**	No ligand in the active site	Cavitation at the nozzle tip	Refill tip to remove bubbles; lower voltage
		Bubbles forming in the ejector	Refill tip to remove bubbles; lower voltage or reduce viscosity
	Buffer showing a negative meniscus	Under-pressure in the line	Increase the reservoir height
	Buffer showing a positive meniscus	Over-pressure in the line	Reduce reservoir height
	Sample no longer ejecting	Bubbles on or near the electrode	Clear bubbles and degas buffer if possible
		Buffer components crystallising at the injector tip	Wet the tip
Spitrobot-2	The puck does not rotate in the puck holder	Rotating parts frozen together	Remove puck, defrost dewar and ensure no water in the mechanism
	The sample changing mechanism does not rotate the puck	Puck is not correctly aligned	Lower the puck and realign
	Shutter refuses to open	Ice or residue is jamming the shutter	Clean shutter lid with 20% EtOH; request opening and closing of shutter; wipe away EtOH and repeat until the shutter starts to move
	Camera images are frozen	Cameras have disconnected	Restart LabVIEW code
	Releasing the triggers during vitrification	Human error	Remove the pin and discard the sample; reset the *Spitrobot*
	Releasing the triggers before vitrification	Human error	Provided no ligand has been deposited, clear the error state and restart
	Spitrobot in error state, unable to trigger vitrification	LN_2 level too low	Refill LN_2
	Spitrobot failed to release pin after vitrification	Failure of electronic release	Remove loop and clean; reset the *Spitrobot*
	Spitrobot released pin too late, trapped in shutter	Shutter mechanically delayed or timing error	Remove the pin and discard the sample; check shutter and reset the *Spitrobot*
	Pin not accurately placed in the puck	Puck well is misaligned	Remove the pin and discard the sample; re-align puck

REFERENCES

1. Cheng NS. Formula for the Viscosity of a GlycerolWater Mixture. 2008;47(9):3285–3288. doi: 10.1021/ie071349z
2. Edward JT. Molecular volumes and the Stokes-Einstein equation. 1970;47(4):261. doi: 10.1021/ed047p261
3. Haynes WM. *X-Ray Data Collection From Macromolecular Crystals*. CRC Press, 2016.
4. Schmidt M. Mix and inject: Reaction initiation by diffusion for time-resolved macromolecular crystallography. *Advances in Condensed Matter Physics.* 2013;1:167276. doi: https://doi.org/10.1155/2013/167276
5. Elamin K, Swenson J. Brownian motion of single glycerol molecules in an aqueous solution as studied by dynamic light scattering. 2015;91(3):032306. doi: 10.1103/PhysRevE.91.032306
6. Kuo AC. *Poly (dimethylsiloxane)*. CRC Press, 1999.
7. Gennes PGD. *Scaling concepts in polymer physics*. Cornell university Press, 1979.
8. Apostolopoulou V, Junius N, Sear RP, Budayova-Spano M. Mixing Salts and Poly(ethylene glycol) into Protein Solutions: The Effects of Diffusion across Semipermeable Membranes and of Convection. 2020;20(6):3927–3936. doi: 10.1021/acs.cgd.0c00246
9. Han F, Zhang J, Chen G, Wei X. Density, Viscosity, and Excess Properties for Aqueous Poly(ethylene glycol) Solutions from (298.15 to 323.15) K. 2008;53(11):2598–2601. doi: 10.1021/je800464t
10. Butryn A, Simon PS, Aller P, et al. An on-demand, drop-on-drop method for studying enzyme catalysis by serial crystallography. *Nature communications.* 2021;12(1):4461. doi: doi.org/10.1038/s41467-021-24757-7
11. Mehrabi P, Schulz EC, Agthe M, et al. Liquid application method for time-resolved analyses by serial synchrotron crystallography. *Nature methods.* 2019;16(10):979–982. doi: 10.1038/s41592-019-0553-1
12. Bar-Even A, Noor E, Savir Y, et al. The moderately efficient enzyme: evolutionary and physicochemical trends shaping enzyme parameters. *Biochemistry.* 2011;50(21):4402–4410. doi: doi: 10.1021/bi2002289
13. Russi S, Juers DH, Sanchez-Weatherby J, et al. Inducing phase changes in crystals of macromolecules: Status and perspectives for controlled crystal dehydration. *J. Struct. Biol.* 2011;175(2):236–243. doi: 10.1016/j.jsb.2011.03.002
14. Cipriani F, Felisaz F, Launer L, et al. Automation of sample mounting for macromolecular crystallography. *Acta Crystallogr D Biol Crystallogr.* 2006;62:1251-1259. doi: 10.1107/S0907444906030587
